# Anionic and Magnetic Ordering in Rare Earth Tantalum Oxynitrides with an *n* = 1 Ruddlesden–Popper Structure

**DOI:** 10.1021/acs.chemmater.4c00533

**Published:** 2024-05-08

**Authors:** Jhonatan R. Guarín, Carlos Frontera, Judith Oró-Solé, Bastian Colombel, Clemens Ritter, François Fauth, Josep Fontcuberta, Amparo Fuertes

**Affiliations:** † Institut de Ciència de Materials de Barcelona (ICMAB-CSIC), Campus UAB, 08193 Bellaterra, Spain; ‡ Institut Laue-Langevin, 71 Av. de Martyrs, BP 156, F-38042 Grenoble Cedex 9, France; § CELLS-ALBA Synchrotron, Barcelona 08290, Spain

## Abstract

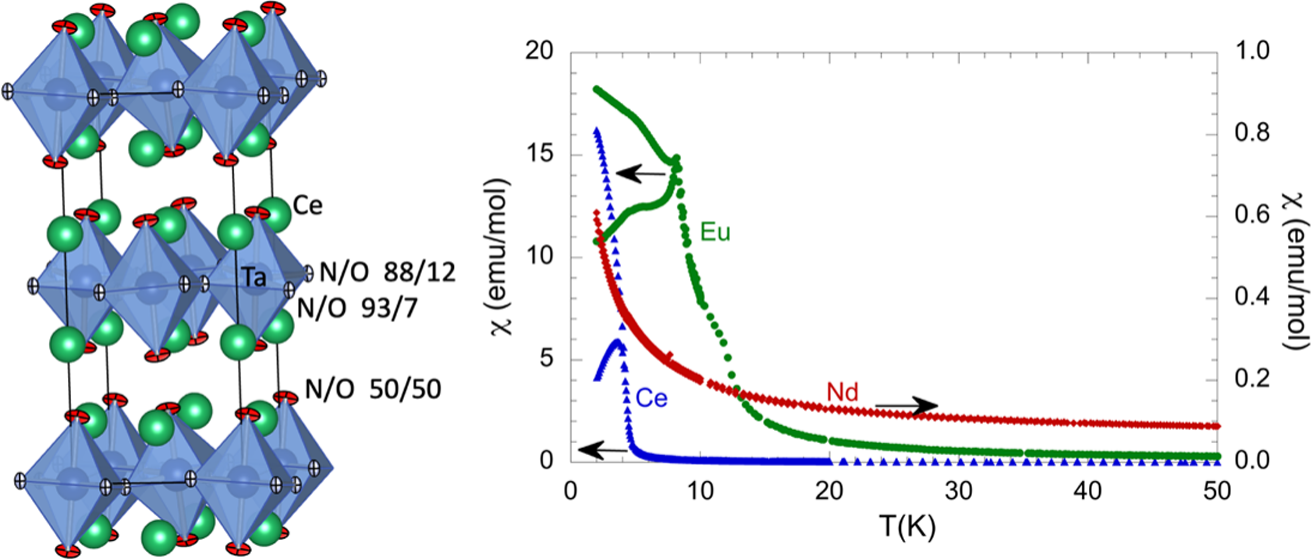

The new compounds R_2_TaO_4–*x*_N_*x*_ with R = La, Ce, Nd, and Eu and 1.20 ≤ *x* ≤ 2.81 have been obtained by a solid-state reaction between metal nitrides and oxides or oxynitrides under N_2_ gas at temperatures between 1200 and 1700 °C. They are the first examples of rare earth transition metal oxynitrides with an *n* = 1 Ruddlesden–Popper structure and show different anion stoichiometries, crystal structures, and magnetic properties. Synchrotron X-ray powder diffraction and electron diffraction indicate that the lanthanum, cerium, and neodymium compounds crystallize in the orthorhombic space group *Pccn*, with cell parameters *a* = 5.72949(2), *b* = 5.73055(5), and *c* = 12.77917(6) Å for La_2_TaO_1.31_N_2.69_, *a* = 5.70500(5), *b* = 5.71182(4), and *c* = 12.61280(7) Å for Ce_2_TaO_1.19_N_2.81_, and *a* = 5.70466(3), *b* = 5.70476(5), and *c* = 12.32365(5) Å for Nd_2_TaO_1.46_N_2.54_. In contrast, Eu_2_TaO_2.80_N_1.20_ shows a tetragonal *I*4_1_/*acd* superstructure doubling the *c* axis, with parameters *a* = 5.71867(2) and *c* = 25.00092(19) Å. Refinement of neutron powder diffraction data of Ce_2_TaO_1.19_N_2.81_ indicated the nitrogen order in the two equatorial positions of the tantalum octahedron, with refined N/O occupancies of 0.930(7)/0.070 and 0.876(13)/0.124, and the axial position is occupied by 50% of each anion. This anion ordering agrees with the distribution predicted by Pauling’s second crystal rule. Magnetization measurements show that the cerium and europium compounds are ordered magnetically at low temperatures, while the neodymium compound remains paramagnetic down to 2 K, as a consequence of suppression of the effective magnetic moment of the latter when reducing the temperature.

## Introduction

Perovskite oxynitrides have been widely investigated in the last two decades because of their notable applications as electronic and photocatalytic materials. The strategy for finding new properties is based on the lower electronegativity of nitrogen compared to that of oxygen, which induces changes in the electronic structures and increases the covalency of bonds with the metals. Additionally, the larger electrical charge of the anion N^3–^ compared to O^2–^ allows the formation of phases with new combinations of cations that show oxidation states different than in the analogous oxides. The majority of known compounds are pseudocubic simple perovskites derived from the *Pm3̅m* aristotype, of the general formula ABO_3–*x*_N_*x*_ with A = alkaline earth or rare earth metal and B = early transition metal, showing different crystal symmetries induced by octahedral tilting and the anion order.^1^ Important examples of materials are nontoxic pigments La_1–*x*_Ca_*x*_TaO_1+*x*_N_2–*x*_,^2^ EuNbO_2_N and EuWO_1+*x*_N_2–*x*_ with colossal magnetoresistance at low temperatures,^3,4^ BaTaO_2_N and SrTaO_2_N with high dielectric constants,^5^ and several tantalum perovskites with photocatalytic activity in water splitting.^6,7^

There are few reported perovskite oxynitrides with complex structures. Examples of double and triple perovskites are Sr_2_FeMoO_4.9_N_1.1_,^8^ Sr_2_FeWO_5_N,^9^ La_2_MnTaO_5_N,^10^ and Eu_3_Ta_3_O_3.66_N_5.34_,^11^ all of them showing magnetic ordering at low temperatures. Polar BaWON_2_ is the only known example of a hexagonal perovskite.^12^ Layered, Ruddlesden–Popper^13^ perovskite oxynitrides (AX)(ABX_3_)*_n_* (X = O, N) were first reported by R.Marchand and co-workers for the *n* = 1 members Sr_2_TaO_3_N, Ba_2_TaO_3_N,^14^ and R_2_AlO_3_N (R = La, Nd, Sm).^15^ We prepared the *n* = 1 and 2 members of the series (SrO)(SrNbO_2_N)*_n_*, with the compositions Sr_2_NbO_3_N and Sr_3_Nb_2_O_5_N_2_, respectively,^16^ and the *n* = 2 compound Eu_3_Ta_2_O_3_N_4_ has been recently reported.^17^

Rare earth perovskite oxynitrides are known for the transition metals Cr, Ti, Zr, Hf, V, Nb, Ta, and W, and they have been mostly investigated for their electronic and photocatalytic properties. RCrO_3–*x*_N_*x*_ (R = La, Pr, and Nd) perovskites show antiferromagnetic coupling of Cr^3+^/Cr^4+^ spins with Neel temperatures from 285 to 214 K.^18^ Vanadium perovskites with R = La and Pr show spin freezing transitions at low temperatures.^19^ EuTaO_2_N, EuNbO_2_N,^3^ and EuWO_1+*x*_N_2–*x*_^4^ are ferromagnetic with *T*_c_ values between 5 and 12 K because of Eu^2+^ spin ordering. LaTiO_2_N^20^ and RHfO_2_N^21^ (R = La, Nd, Sm) compounds are visible light-active photocatalysts in water oxidation and reduction, whereas LaTaON_2_, in addition to a photocatalyst for water splitting,^22^ is a high-dielectric permittivity material.^23^

In this paper, we report the synthesis, crystal structures, and magnetic properties of the new compounds R_2_TaO_4–*x*_N_*x*_ (R = La, Ce, Nd, and Eu) that are the first examples of transition metal *n* = 1 Ruddlesden–Popper oxynitrides with a rare earth cation at the A sites. These oxynitrides can be stabilized by using a high-temperature synthesis method under N_2_, starting with a mixture of metal nitrides and oxides. The obtained anion stoichiometries indicate reduction of the cations during synthesis and are determined by the stable oxidation states of tantalum (Ta^4+^, Ta^5+^) and the rare earth cations (La^3+^, Ce^3+^, Nd^3+^, and Eu^2+^/Eu^3+^) under the preparative conditions. The anion distribution is investigated by neutron diffraction for Ce_2_TaO_1.19_N_2.81_, showing the order of nitrides at the equatorial sites of the octahedra, whereas the axial positions are occupied by 50% of each anion. The cerium and europium compounds develop low-temperature (<10 K) magnetic ordering, while the Nd compound is paramagnetic down to 2 K. The striking differences between the magnetic behaviors of the Ce^3+^, Nd^3+^, and Eu^2+^/Eu^3+^ compounds are rationalized in terms of the distinct role that crystal field effects and exchange interactions play in determining the singlet or triplet ground state of the rare earth cations.

## Experimental Methods

### Synthesis and Chemical Characterization

Samples of 100 to 200 mg of R_2_TaO_4–*x*_N_*x*_ (R = La, Ce, Nd, Eu) compounds were prepared by a solid-state reaction under N_2_ gas (Air Liquide, 99.9999%) at temperatures between 1200 and 1700 °C, starting from mixtures with different proportions of RN, R_2_O_3_, Ta_3_N_5_, and TaON, while keeping the stoichiometric ratio R/Ta of 2:1. La_2_TaO_1.31_N_2.69_ was prepared starting with LaN and Ta_3_N_5_ (Alfa Aesar 99.9%) in a molar ratio of 6:1 at 1700 °C, Ce_2_TaO_1.19_N_2.81_ was prepared from CeN, Ta_3_N_5_, and TaON in a molar ratio of 6:0.375:1.875 at 1500 °C, Nd_2_TaO_1.46_N_2.54_ was prepared from Nd_2_O_3_(Aldrich 99.99%), NdN (Alfa Aesar 99.9%), and Ta_3_N_5_ at 1500 °C with a ratio of 0.05:1.9:0.33, and Eu_2_TaO_2.80_N_1.20_ was obtained from Eu_2_O_3_ (Sigma-Aldrich 99.9%), EuN (Materion, 99.9%), and Ta_3_N_5_ in a ratio of 0.85:0.30:0.33 at 1200 °C. The proportion of the reactants determining the O/N ratio in the initial mixture and the maximum synthesis temperature were optimized from several syntheses performed for each compound, until the sample was a single phase from laboratory X-ray diffraction. CeN was obtained by treatment under N_2_ of Ce chips (Strem 99.9%) at 1000 °C. Ta_3_N_5_ was obtained from Ta_2_O_5_ (Sigma-Aldrich 99.99%) by treatment under NH_3_ (Carburos Metálicos 99.9%) at 880 °C using a flow rate of 600 cm^3^/min and several treatments of 15 h with intermediate regrinding. TaON was prepared by a similar procedure but using a flow rate of 40 cm^3^/min and two treatments of 3 h with intermediate regrinding. Nd_2_O_3_ and Eu_2_O_3_ were treated at 900 °C under a dynamic vacuum of 1 × 10^–3^ Torr for dehydration. Handling, mixing, and pelletizing of the reactants were performed inside a glovebox under recirculating Ar. The samples were placed in molybdenum crucibles covered by Zr foil that was also placed in a second molybdenum crucible in order to scavenge oxygen and water from the N_2_ gas. The reaction tube was evacuated to 10^–3^ Torr and purged several times with N_2_ before starting the thermal cycle. This consisted of a single treatment of heating at 300 °C/h up to the maximum temperature that was kept for 3 h, with further natural cooling to room temperature.

Nitrogen contents were determined by combustion analysis performed in a Thermo Fisher Scientific instrument, heating the samples in oxygen up to 1060 °C and using MgO, WO_3_, and Sn as additives and atropine as a reference standard. EDX analyses of cation contents were performed in a FEI Quanta 200 FEG microscope equipped with an EDAX detector with an energy resolution of 132 eV. The analyses were performed on 10–15 crystallites for each sample.

### Structural Characterization

Laboratory powder X-ray diffraction was used for controlling the purity of the samples during the synthesis. Data were acquired on a Panalytical X′Pert Pro MPD diffractometer using Cu Kα radiation (λ = 1.5418 Å) and on a Bruker D8 Advance A25 diffractometer in a Debye–Scherrer configuration with Mo Kα_1_ radiation (λ = 0.7093 Å) using capillary samples (0.3 mm diameter). High-angular resolution synchrotron X-ray powder diffraction data were collected at room temperature from capillary samples in the angular range of 2.0° ≤ 2θ ≤ 56.9° at the MSPD beamline^24^ of the ALBA Synchrotron (Cerdanyola del Vallès, Spain), using 30 keV energy that resulted in exact wavelengths of 0.4137, 0.4139 and 0.4142 Å as determined by refining the SRM640d NIST Si standard. Neutron powder diffraction was used to determine the anion distribution in Ce_2_TaO_1.19_N_2.81_. Data on a 80 mg sample were collected for 19 h at room temperature on the high-intensity D20 diffractometer at the Institut Laue-Langevin (ILL), France, using a vanadium can as a sample holder. The pattern was measured in scanning mode with a short wavelength of 1.37 Å created by using 118° takeoff angle, giving high resolution. Rietveld analysis was carried out using the program Fullprof.^25^ Background refinement was performed by linear interpolation, and data were corrected from absorption.

Electron diffraction micrographs were obtained in a JEOL 1210 transmission electron microscope operating at 120 kV using a side entry double tilt ±60°/±30° specimen holder. The samples were prepared by depositing the powder on a copper grid coated with a holey carbon film.

### Magnetic Measurements

Magnetic measurements were performed at fields of 25 and 10 kOe between 2 and 300 K using a Quantum Design SQUID magnetometer. Magnetization field loops were measured between −70 and +70 kOe between 2 and 16 K.

## Results and Discussion

### Synthesis and Crystal Structures of R_2_TaO_4–*x*_N_*x*_ Compounds

The synthesis of new rare earth tantalum *n* = 1 Ruddlesden–Popper compounds was performed using the reactions at high temperature under N_2_
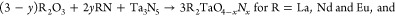
1
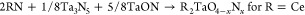
2

The best samples of La, Nd, and Eu were obtained for *y* = 3, 2.85, and 0.45, respectively. In the case of cerium, we used TaON as an oxygen source because Ce_2_O_3_ is not easily available commercially. This synthetic route is similar to that used for the preparation of the rare earth perovskites LaTaON_2_,^23^ EuTaO_2.37_N_0.63_, and Eu_3_Ta_3_O_3.66_N_5.34_^11^ that we have recently reported and produces samples of high crystallinity with short reaction times. The rare earth perovskite oxynitrides are commonly prepared under NH_3_ at temperatures below 1000 °C, starting with oxide precursors such as scheelites because the rare earth binary oxides are poorly reactive in these conditions.^26^ R_2_O_3_ reactants have been used for the synthesis of few compounds under NH_3_ but in the presence of fused salts that increase the kinetics of the nitridation^27^ or in high-pressure conditions.^28^ The combination of rare earth nitrides, RN, with different proportions of oxides and nitrides allows one to change the nitrogen/oxygen ratio in the initial mixture up to avoiding or minimizing the impurity phases. These are frequently formed in the synthesis of nitrides at high temperatures because of the presence of oxygen or water around the sample.

The black-colored R_2_TaO_4–*x*_N_*x*_ samples were prepared at different temperatures optimized in each case, from 1200 °C for R = Eu to 1700 °C for R = La, and showed analyzed nitrogen contents of 2.69(3), 2.81(3), 2.54(3), and 1.20(3) atoms per formula for R = La, Ce, Nd, and Eu, respectively. In all cases, these contents were below the nitrogen stoichiometry in the mixture of reactants (3, 3.25, 3.56, and 1.96 for La, Ce, Nd, and Eu samples, respectively), indicating the incorporation of extra oxygen in the samples during synthesis. Nitrogen loss at high temperatures has been observed in other tantalum perovskites such as SrTaO_2_N^29^ and LaTaON_2_^23^ and has been interpreted as a decomposition reaction releasing N_2_ with partial reduction of Ta^5+^ to Ta^4+^, analogous to the oxygen loss of transition metal oxides at high temperatures that produces reduced oxides together with O_2_.^30^ The corresponding cation ratios determined by EDX were La/Ta = 1.82(17), Ce/Ta = 1.81(10), Nd/Ta = 1.77(17), and Eu/Ta = 1.89(15), which agree with the nominal compositions within the experimental error. The oxygen stoichiometries were calculated by difference, assuming that the total anion content was four atoms per formula, resulting in La_2_TaO_1.31_N_2.69_, Ce_2_TaO_1.19_N_2.81_, Nd_2_TaO_1.46_N_2.54_, and Eu_2_TaO_2.80_N_1.20_. Considering charge compensation and the trivalent oxidation state for the rare earth cations, the nitrogen deficiency with respect to the ideal R_2_TaON_3_ composition in the La, Ce, and Nd compounds would result in a proportion of Ta^4+^ of 31, 19, and 46%, respectively. Compared with the other rare earth compounds, the observed N content in Eu_2_TaO_2.80_N_1.20_ indicates that europium is dominantly divalent, which is consistent with the observed structural data and the magnetic properties (see below). In the synchrotron X-ray powder diffraction patterns of lanthanum, neodymium, and europium samples, we detected the perovskite-phase RTaON_2_ with the respective amounts of 6.6, 5.1, and 1.9% (w/w) as determined from Rietveld refinement.

**Figure 1:**
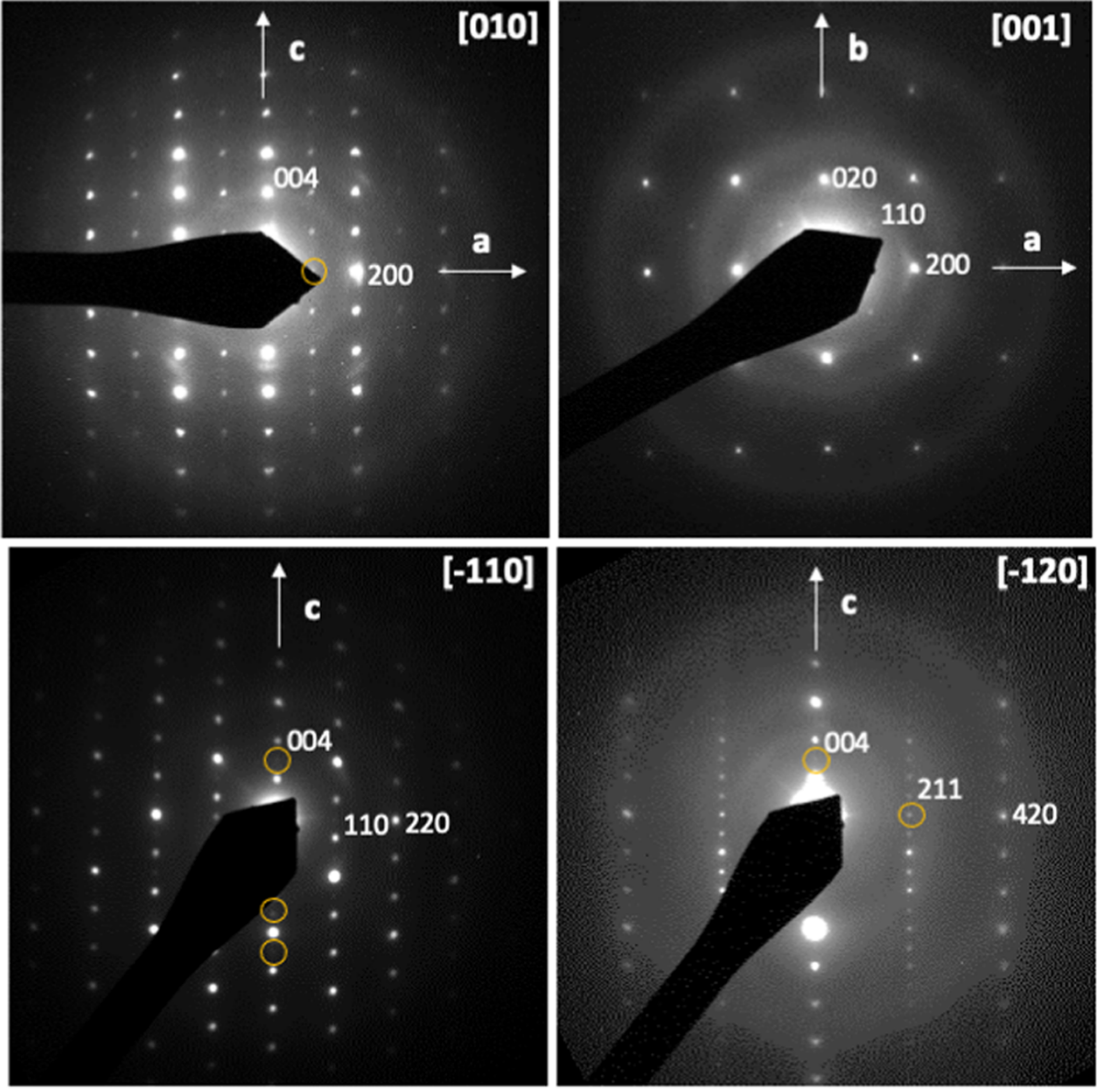
Selected electron diffraction patterns of Ce_2_TaO_1.19_N_2.81_. Yellow circles indicate multiple diffraction reflections.

**Figure 2:**
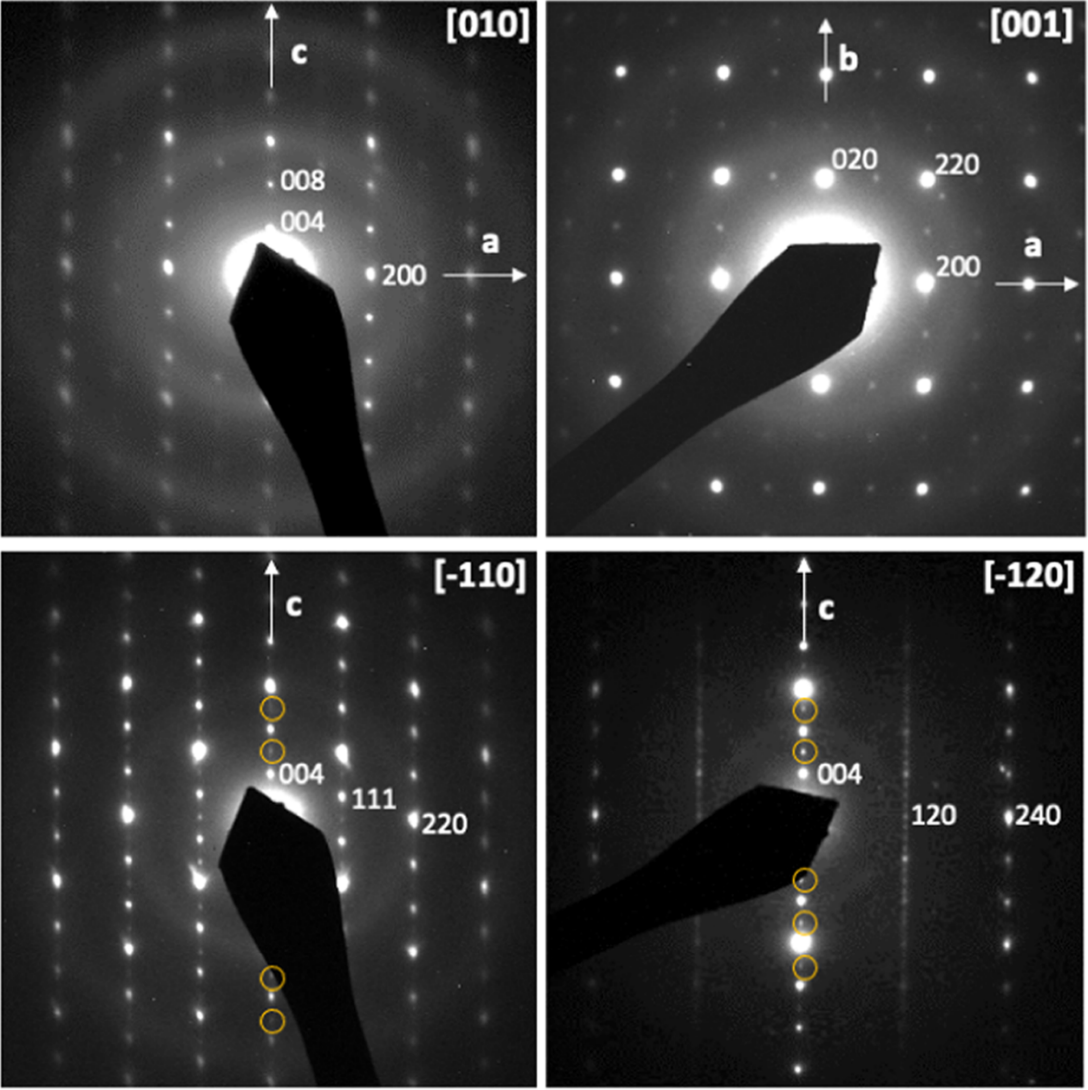
Selected electron diffraction patterns of Eu_2_TaO_2.80_N_1.20_ with reflections indexed in the tetragonal cell with *a* ≃ 5.7 and *c* ≃ 25 Å. Yellow circles indicate multiple diffraction reflections.

The electron diffraction patterns of the compounds La_2_TaO_1.31_N_2.69_, Ce_2_TaO_1.19_N_2.81_, and Nd_2_TaO_1.46_N_2.54_ showed additional reflections to those expected for the *I*4/*mmm* space group of the K_2_NiF_4_ aristotype (see Figures 1, S1, and S2) indicative of a tilted superstructure with parameters √2a_0_ × √2a_0_ × *c*_0_ (where *a*_0_ and *c*_0_ are the parameters of the *I*4/*mmm* cell). The reconstruction of the reciprocal lattice led to an orthorhombic cell with parameters *a*, *b* ≃ 5.7 Å and the respective *c* axis of 12.89, 12.60, and 12.53 Å for R = La, Ce, and Nd, with the observed reflection conditions consistent with the space group *Pccn* (No. 56) (*hk*0*, h*+*k* = 2*n; h*0*l, l* = 2*n;* 0*kl, l* = 2*n; h*00*, h* = 2*n;* 0*k*0*, k* = 2*n; 00l, l* = 2*n*). This space group corresponds to a tilted *n* = 1 Ruddlesden–Popper structure with out-of-phase rotations around the a and *b* axis, notated as ϕ_1_ ϕ_2_ 0 for the first layer of octahedra and ϕ_2_ ϕ_1_ 0 for the second layer of octahedra at the origin and body center of the *I*4/*mmm* parent cell, and no rotation around the *c* axis.^31,32^ In contrast, the compound Eu_2_TaO_2.80_N_1.20_ showed an additional superstructure along the *c* axis doubling c_0_, with cell parameters *a* = 5.72 Å and *c* = 24.99 Å (Figure 2). The electron diffraction planes indicated reflection conditions compatible with the space group *I*4_1_/*acd*, which has been reported for K_2_NiF_4_ compounds including Sr_2_IrO_4_.^33^ Weak additional reflections were also observed that could be indexed in a larger cell, with *a* = 8.15 Å and *c* = 24.99 Å.

**Figure 3:**
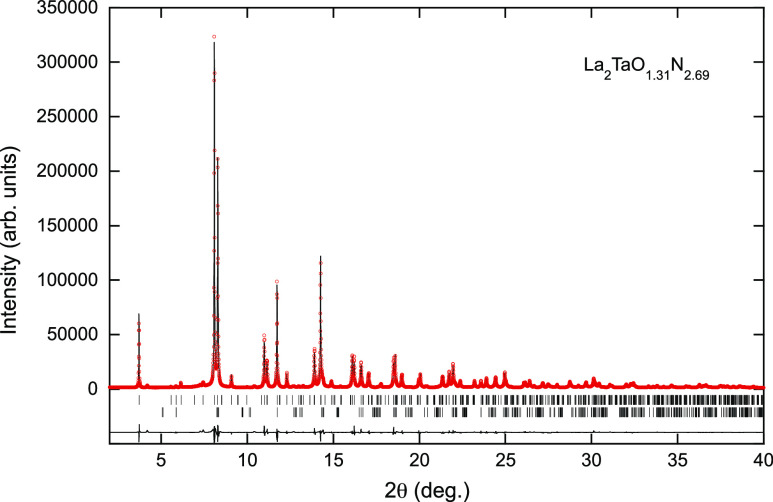
Rietveld fit to the synchrotron X-ray powder diffraction pattern of La_2_TaO_1.31_N_2.69_ performed in the space group *Pccn* with parameters *a* = 5.72949(2), *b* = 5.73055(5), and *c* = 12.77917(6) Å. Upper and lower reflection markers are, respectively, for La_2_TaO_1.31_N_2.69_ and LaTaON_2_.^23^

**Figure 4:**
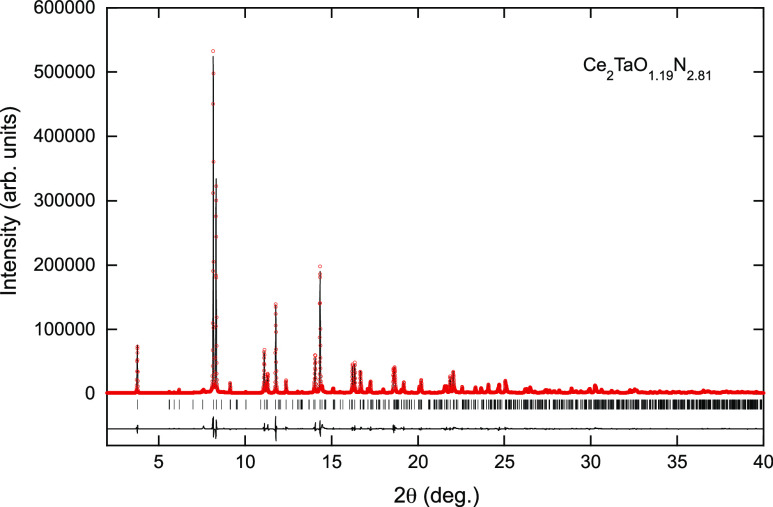
Rietveld fit to the synchrotron X-ray powder diffraction pattern of Ce_2_TaO_1.19_N_2.81_ performed in the space group *Pccn* with cell parameters *a* = 5.70500(5), *b* = 5.71182(4), and *c* = 12.61280(7) Å.

**Figure 5:**
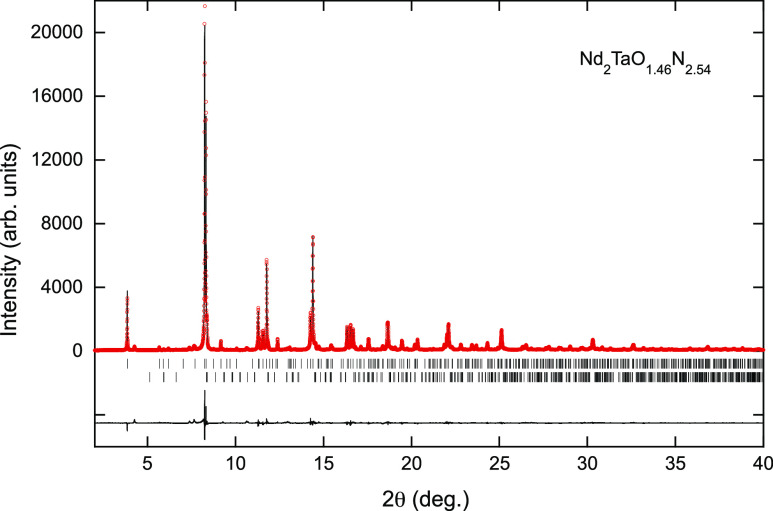
Rietveld fit to the synchrotron X-ray powder diffraction pattern of Nd_2_TaO_1.46_N_2.54_ performed in the space group *Pccn* with cell parameters *a* = 5.70466(3), *b* = 5.70475(5), and *c* = 12.32366(5) Å. Upper and lower reflection markers are, respectively, for Nd_2_TaO_1.46_N_2.54_ and NdTaON_2_.

**Figure 6:**
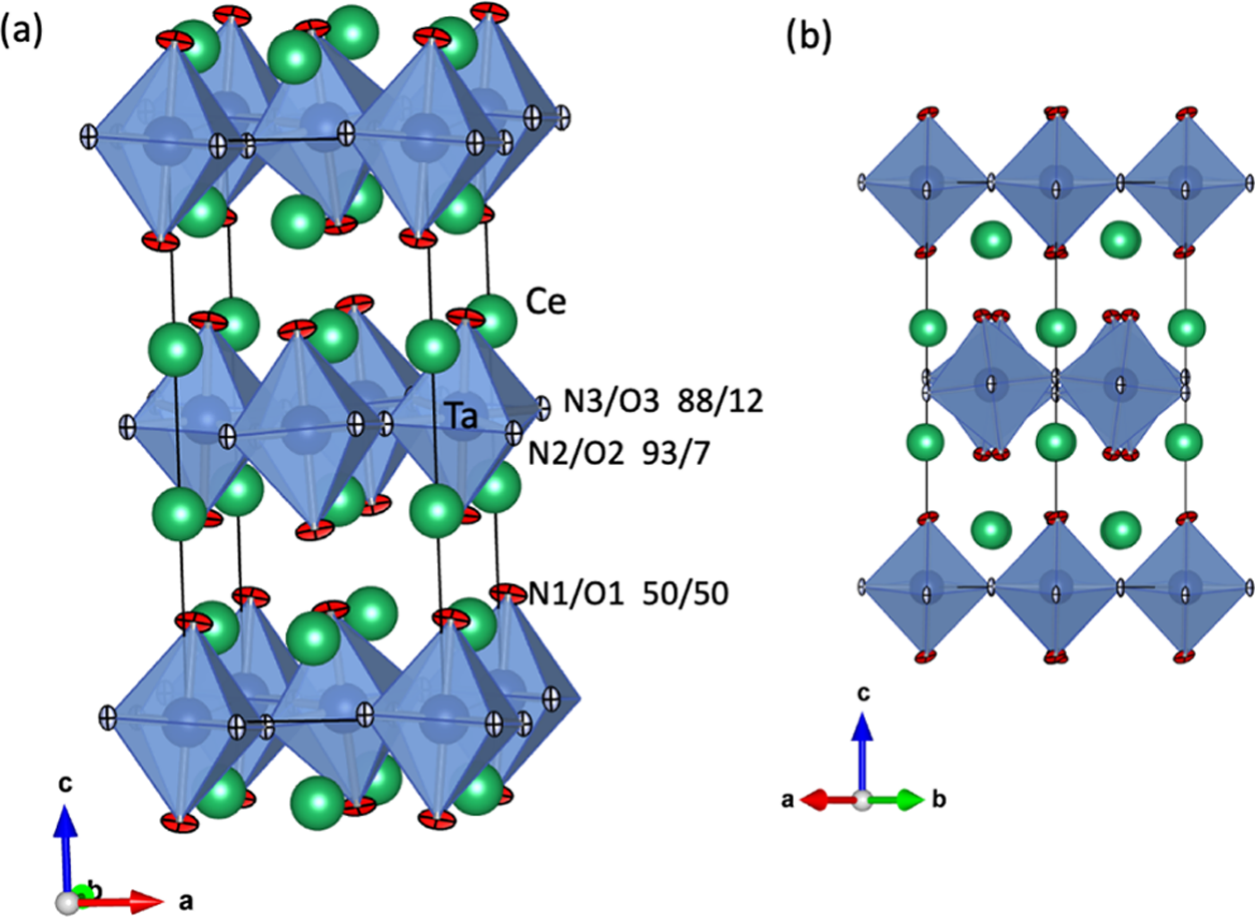
(a) Crystal structure of Ce_2_TaO_1.19_N_2.81_ determined from neutron powder diffraction data. Thermal ellipsoids of the anions are shown at 50% probability. The refined N/O population (%) is indicated for each anion site. (b) Projection along 110 showing the tilting of the Ta octahedra.

Rietveld refinement of synchrotron X-ray diffraction data was performed in the *Pccn* space group for the La, Ce, and Nd compounds (Figures 3, 4, and 5 and Tables 1, 2, and S1, respectively) and led the cell parameters *a* = 5.72949(2), *b* = 5.73055(5), and *c* = 12.77917(6) Å for La_2_TaO_1.31_N_2.69_, *a* = 5.70500(5), *b* = 5.71182(4), and *c* = 12.61280(7) Å for Ce_2_TaO_1.19_N_2.81_, and *a* = 5.70466(3), *b* = 5.70475(5), and *c* = 12.32365(5) Å for Nd_2_TaO_1.46_N_2.54_. For the refinement of the La and Nd compounds, we fixed a statistical distribution of nitrogen and oxygen in the three available anion sites because the X-rays do not provide enough contrast between the two anions. For Ce_2_TaO_1.19_N_2.81_, we used and fixed the occupancies obtained from the refinement of neutron diffraction data (see the next section). The structural model of this compound is shown in Figure 6.

**Table 1:** Summary of the *Pccn* Model Refined against Room-Temperature Synchrotron Powder X-ray Diffraction Data for La_2_TaO_1.31_N_2.69_ (λ = 0.4137 Å)^a^^,^^b^

atom	site	*x*	*y*	*z*	*B* (Å^2^)	occupancy
La	*8e*	0.4965(7)	0.0043(6)	0.1419(5)	0.735(14)	1
Ta	*4a*	0	0	0	1.000(18)	1
O1/N1	*8e*	0.0185(13)	0.0600(6)	0.16527(18)	0.401(16)	0.33/0.67
O2/N2	4c	0.25	0.25	0.4761(5)	0.401	0.33/0.67
O3/N3	*4d*	0.25	0.75	–0.0065(4)	0.401	0.33/0.67

bond	*d* (Å)	bond	*d* (Å)	bond	*d* (Å)
Ta–O1,N1	2.142(2) × 2	Ta–O2,N2	2.049(1) × 2	Ta–O3,N3	2.0276(2) × 2
La–O1,N1	2.495(3)	La–O1,N1	2.516(5)	La–O1,N1	2.773(8)
La–O1,N1	3.023(8)	La–O1,N1	3.249(5)	La–O2,N2	2.551(5)
La–O2,N2	2.910(5)	La–O3,N3	2.662(4)	La–O3,N3	2.778(4)

a Refined cell parameters and agreement factors are *a* = 5.72949(2), *b* = 5.73055(5), and *c* = 12.77917(6) Å. *V* = 419.580(4) Å^3^. *R*_Bragg_ = 3.46% and *R*_wp_ = 7.16%.

b The O/N occupancies were fixed to a statistical distribution considering the chemical analysis. The temperature factors were common for the three anions sites. Average bond distances: Ta–O,N 2.073 Å and La–O,N 2.773 Å.

**Table 2:** Summary of the *Pccn* Model Refined against Room-Temperature Synchrotron X-ray Powder Diffraction Data for Nd_2_TaO_1.46_N_2.54_ (λ = 0.4139 Å)^a^^,^^b^

atom	site	*x*	*y*	*z*	*B* (Å^2^)	occupancy
Nd	*8e*	0.5118(4)	–0.00580(8)	0.14307(3)	0.868(2)	1
Ta	*4a*	0	0	0	1.039(10)	1
O1/N1	*8e*	–0.047(4)	–0.049(4)	0.1774(7)	1.41(13)	0.37/0.63
O2/N2	4c	0.25	0.25	0.5277(7)	1.41	0.37/0.63
O3/N3	*4d*	0.25	0.75	–0.013(3)	1.41	0.37/0.63

bond	*d* (Å)	bond	*d* (Å)	bond	*d* (Å)
Ta–O1,N1	2.220(9) × 2	Ta–O2,N2	2.046(3) × 2	Ta–O3,N3	2.023(3)(×2)
Nd–O1,N1	2.259(10)	Nd–O1,N1	2.56(2)	Nd–O1,N1	2.58(2)
Nd–O1,N1	3.20(3)	Nd–O1,N1	3.23(3)	Nd–O2,N2	2.526(12)
Nd–O2,N2	2.866(15)	Nd–O3,N3	2.56(2)	Nd–O3,N3	2.81(3)

a Refined cell parameters and agreement factors: *a* = 5.70466(3), *b* = 5.70475(5), and *c* = 12.32365(5) Å. *V* = 401.056(4) Å^3^. *R*_Bragg_ = 4.74% and *R*_wp_ = 9.19%.

b The O/N occupancies were fixed to a statistical distribution considering the chemical analysis. The temperature factors were common for the three anions sites. Average bond distances: Ta–O,N 2.096 Å and Nd–O,N 2.732 Å.

**Figure 7:**
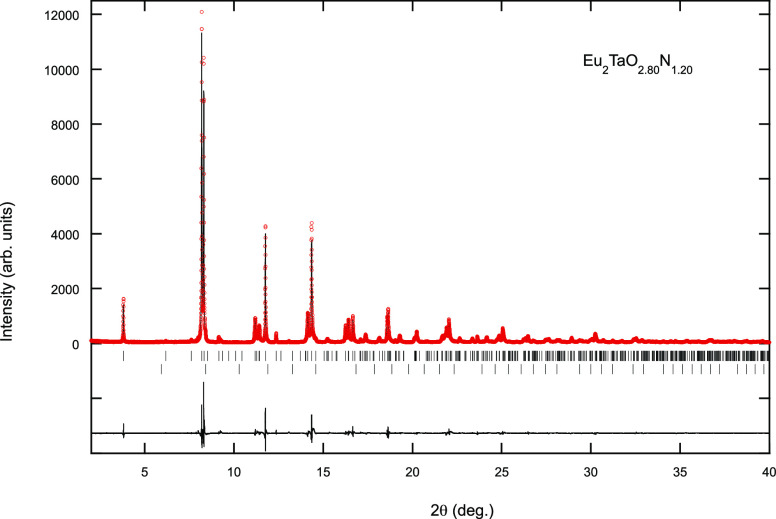
Rietveld fit to the synchrotron X-ray powder diffraction pattern of Eu_2_TaO_2.80_N_1.20_ performed in the space group *I*4_1_/*acd* with cell parameters *a* = 5.71867(2) and *c* = 25.00092(19) Å. Upper and lower reflection markers are, respectively, for Eu_2_TaO_2.80_N_1.20_ and EuTaO_2_N, respectively.

**Figure 8:**
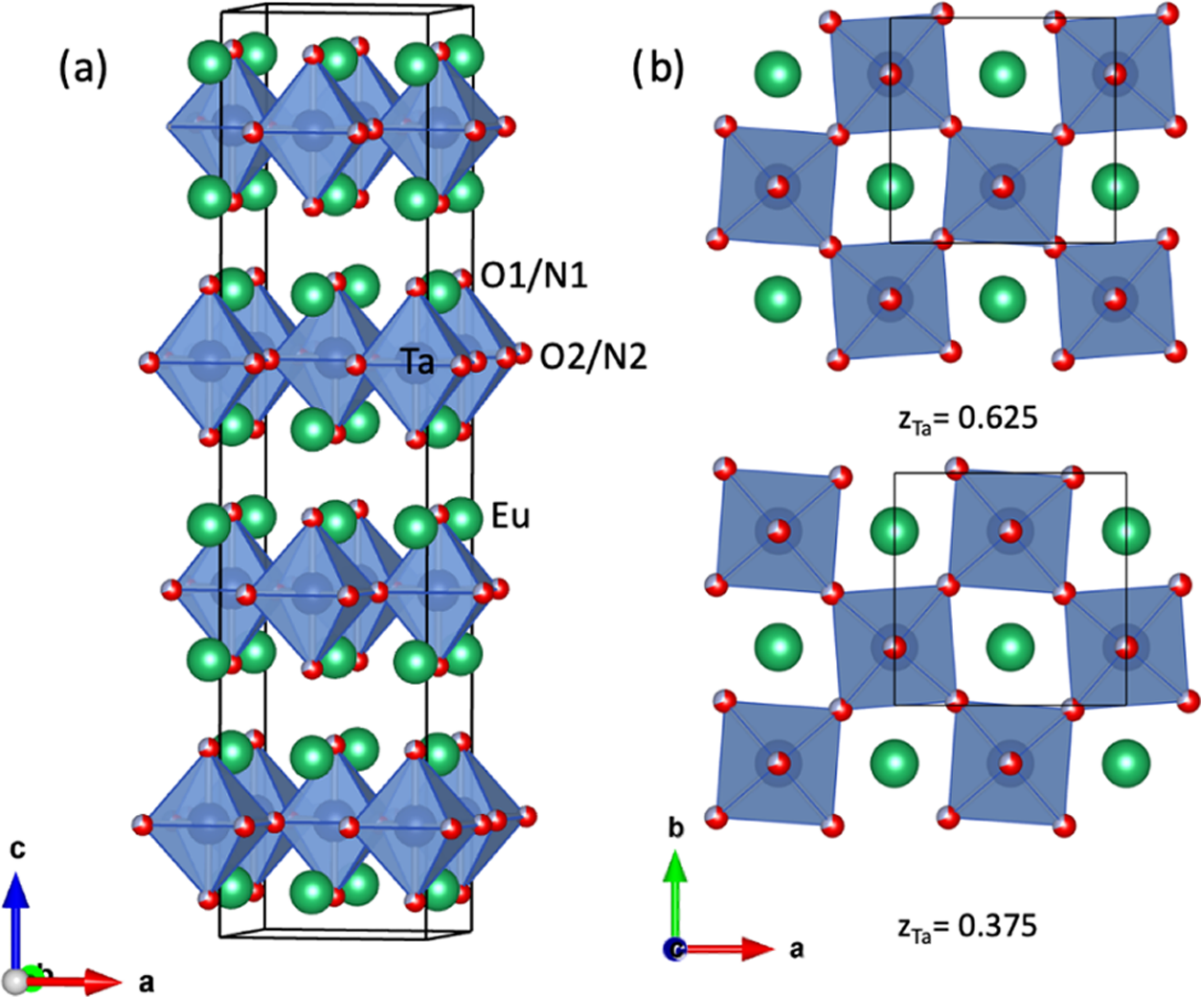
(a) Crystal structure of Eu_2_TaO_2.80_N_1.20_ determined from synchrotron powder X-ray diffraction data. The nitride and oxide anions are depicted as gray and red spheres, respectively, and are distributed statistically in the X1 and X2 sites with proportions according to the chemical analysis. (b) Projection along c of the two central layers of the unit cell, showing the tilting of the Ta octahedra.

The refinement of the crystal structure of Eu_2_TaO_2.80_N_1.20_ from synchrotron X-ray diffraction data was performed in the space group *I*4_1_/*acd* starting with the atomic coordinates of Sr_2_IrO_4_^33^ and led to the cell parameters *a* = 5.71867(2) and 25.00092(19) Å (√2*a*_0_ × √2*a*_0_ × 2*c*_0_) (Figures 7 and 8, Table 3). In the refinements of the La and Nd compounds, we fixed a statistical distribution of nitrogen and oxygen in the available anion sites because the X-rays do not provide enough contrast between the two anions. Attempts to refine a model with the larger cell of *a* = 8.15 and *c* = 24.99 Å observed by electron diffraction led to chemically inconsistent bond distances and angles. In the *I*4_1_/*acd* model, the doubling of the *c*_0_ parameter results from a sequence of tilts along the *c* axis (θ tilts) that repeats every four layers of octahedra (Figure 8).^31^ The cell volume of this compound normalized to a √2*a*_0_ × √2*a*_0_ × *c*_0_ cell is 408.805 Å^3^, that is larger than for Nd_2_TaO_1.46_N_2.54_ (401.056(4) Å^3^) as a consequence of the divalent state of Eu^2+^, with a larger ionic radius than that of Nd^3+^ (for CN = IX, *r*(Eu^2+^) = 1.30 Å and *r*(Nd^3+^) = 1.163 Å).^34^ The cell volumes of the La (419.580(4) Å^3^) and Ce (411.000(5) Å^3^) compounds are significantly larger than for the Nd phase, as expected from the ionic radii of R^3+^ cations.^34^ The Goldschmidt tolerance factors (*t*) for the four phases have been calculated from the ionic radii considering the formal compositions La_2_^3+^Ta_0.31_^4+^Ta_0.69_^5+^O_1.31_N_2.69_, Ce_2_^3+^Ta_0.19_^4+^Ta_0.81_^5+^O_1.19_N_2.81_, Nd_2_^3+^Ta_0.46_^4+^Ta_0.54_^5+^O_1.46_N_2.54_, and Eu_1.80_^2+^Eu_0.20_^3+^TaO_2.80_N_1.20_, leading to the values of 0.904 (La), 0.896 (Ce), 0.886 (Nd), and 0.930 (Eu), respectively. The larger *t* of the europium compound, together with the lower nitride content of this phase and an expected distinct anion ordering (see the next section), may account for its different crystal symmetry compared with the other rare earth derivatives.

**Table 3:** Summary of the *I*4_1_/*acd* Model Refined against Room-Temperature Synchrotron X-ray Powder Diffraction Data for Eu_2_TaO_2.80_N_1.20_ (λ= 0.4142 Å)^a^^,^^b^

atom	site	*x*	*y*	*z*	*B* (Å^2^)	occupancy
Eu	1*6d*	0	0.25	0.552190(18)	0.686(10)	1
Ta	*8a*	0	0.25	0.375	0.922(15)	1
O1/N1	*16d*	0	0.25	0.4564(3)	2.816	0.70/0.30
O2/N2	*16f*	0.232(2)	0.482(2)	0.125	2.816	0.70/0.30

bond	*d* (Å)	bond	*d* (Å)
Ta–O1,N1	2.035(8) × 2	Ta–O2,N2	2.027(11) × 4
Eu–O1,N1	2.395(8)	Eu–O1,N1	2.867(1) × 4
Eu–O2,N2	2.830(9) × 2	Eu–O2,N2	2.614(8) × 2

a Refined cell parameters and agreement factors are *a* = 5.71867(2) and *c* = 25.00092(19) Å. *V* = 817.609(8) Å^3^. *R*_Bragg_ = 4.19% and *R*_wp_ = 10.5%.

b Isotropic thermal parameters of the anions were fixed to 2.816 Å^2^. O/N occupancies were fixed to a statistical distribution considering the chemical analysis. Average bond distances: Ta–O,N 2.030 Å and Eu–O,N 2.750 Å.

### Neutron Diffraction Study of Ce_2_TaO_1.19_N_2.81_: Anion Order and the Prediction of Pauling’s Second Crystal Rule in *n* = 1 Ruddlesden–Popper Oxynitrides

**Figure 9:**
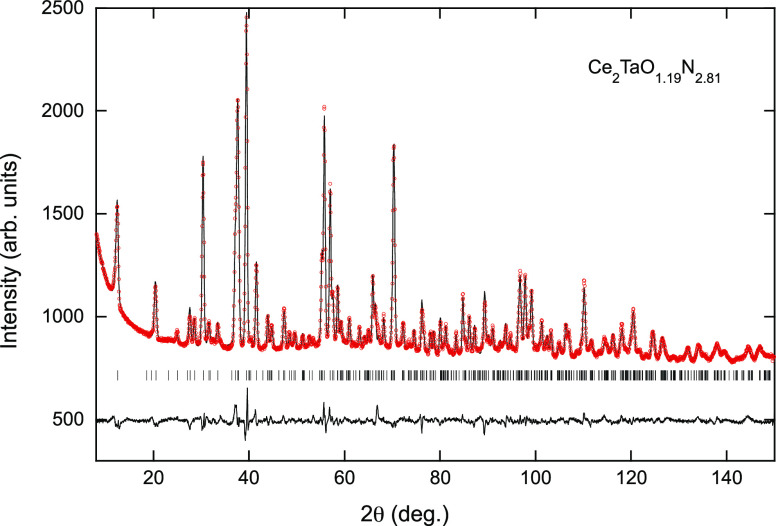
Rietveld fit to the neutron powder diffraction pattern of Ce_2_TaO_1.19_N_2.81_ performed in the space group *Pccn* with cell parameters *a* = 5.75284(19), *b* = 5.75620(15), and *c* = 12.71338(18) Å.

The refinement of neutron diffraction data of Ce_2_TaO_1.19_N_2.81_ (Figure 9) was performed starting with a random distribution of nitrogen and oxygen in the three anion positions of the *Pccn* model, considering full occupancy in all sites and constraining the total content of each anion to the composition determined by chemical analysis. The refined N/O populations for the two equatorial sites were 93/7 (X2) and 88/12 (X3), whereas the obtained occupancies of the axial site X1 were 50% for each anion (Figure 6 and Table 4). This anion distribution shows a near-100% order of the nitride anions at the equatorial sites of the tantalum octahedra. The bond distances for these positions were significantly shorter (*d*(Ta-X2) = 2.0504(3) Å, *d*(Ta-X3) = 2.0346(2) Å) than for the axial site (*d*(Ta-X1) = 2.1855(19) Å). The elongation of the octahedra along the c direction is an indication of the observed anion order, as the axial sites are occupied by 50% O/50% N, and the covalent character of metal–oxygen bonds is lower than for metal–nitrogen bonds. The large thermal parameter observed for the axial site is indicative of the O/N disorder in this position (see Figure 6). Shorter bond M–N distances compared to M–O bonds have been also observed in the hexagonal perovskite BaWON_2_^12^ that shows the total order of N and O in corner-sharing positions and face-sharing positions of the W^6+^ octahedra. The observed anion order in Ce_2_TaO_1.19_N_2.81_ is different from that previously reported in *n* = 1 Ruddlesden–Popper oxynitrides as a consequence of its larger nitrogen content and higher charge of the A cation. In the less nitrided alkaline earth compounds Sr_2_TaO_3_N,^35,36^ Ba_2_TaO_3_N,^36^ and Sr_2_NbO_3_N^37^ crystallizing in the *I*4/*mmm* space group, the nitride anions also prefer the equatorial sites of the octahedra, but the N/O population for these positions is 50/50, whereas the axial sites are fully occupied by oxygen. These anion distributions agree with the prediction of Pauling’s second crystal rule (PSCR),^38,39^ which states that the electric charge of each anion (*q*) tends to compensate the strength of the electrostatic valence bonds from the cations, according to the equation , where *z_i_* is the electric charge of each cation bonded to a given anionic position and *ν_i_* is its coordination number. The *b* values for the equatorial and axial positions in A_2_BO_3_N compounds (A = Sr^2+^, Ba^2+^; B = Nb^5+^, Ta^5+^) are 2.55 and 1.94, respectively, in close agreement with the charge of the anions occupying these sites (*q* = −2.5 and −2)^37,39^ using the determined distributions from neutron diffraction. For ideal R_2_TaON_3_ compounds, the trivalent rare earth cations increase the calculated sums for the equatorial and axial sites to 3 and 2.5, respectively. In Ce_2_TaO_1.19_N_2.81_, considering 19% of Ta^4+^ and 81% of Ta^5+^, the calculated sums are 2.94 and 2.47 for the equatorial and axial sites, respectively, and the observed anion distribution leads to *q* = −2.93, – 2.88, and −2.5 for X2, X3, and X1 positions, respectively, in excellent agreement with the prediction of PSCR. A similar anion ordering can be expected for the other trivalent rare earth derivatives reported in this work, La_2_TaO_1.31_N_2.69_ and Nd_2_TaO_1.46_N_2.54_. However, for Eu_2_TaO_2.80_N_1.20_, the PSCR-predicted distribution is the same as for the alkaline earth oxynitrides because the europium in this compound is essentially divalent, as the alkaline earth cations. Hence, the expected anion populations at the axial sites would be 50/50 for La_2_TaO_1.31_N_2.69_ and Nd_2_TaO_1.46_N_2.54_, whereas for Eu_2_TaO_2.80_N_1.20_, 100% O occupancy is expected for the same positions. For the analyzed anion compositions in each compound, the expected populations at the equatorial sites would be, respectively, O/N 15/85, 23/77, and 40/60 for the La, Nd, and Eu compounds. Future neutron diffraction experiments are planned to corroborate these predictions.

**Table 4:** Summary of the *Pccn* Model Refined against Room-Temperature Neutron Diffraction Data for Ce_2_TaO_1.19_N_2.81_ (λ = 1.37 Å)^a^,^b^

atom	site	*x*	*y*	*z*	*B* (Å^2^)	occupancy
Ce	*8e*	0.4997(17)	0.0115(8)	0.14118(15)	0.89(3)	1
Ta	*4a*	0	0	0	1.09(3)	1
O1/N1	*8e*	0.0309(11)	0.0401(5)	0.17037(13)	2.524	0.498(5)/0.502
O2/N2	4c	0.25	0.25	0.4800(2)	1.205	0.070(7)/0.930
O3/N3	*4d*	0.25	0.75	0.0011(11)	1.205	0.124(13)/0.876

bond	*d* (Å)	bond	*d* (Å)	bond	*d* (Å)
Ta-O1,N1	2.1855(19) × 2	Ta–O2,N2	2.0504(3) × 2	Ta–O3,N3	2.0346(2) × 2
Ce–O1,N1	2.421(3)	Ce–O1,N1	2.614(5)	Ce–O1,N1	2.727(12)
Ce–O1,N1	3.083(12)	Ce–O1,N1	3.202(5)	Ce–O2,N2	2.591(6)
Ce–O2,N2	2.854(6)	Ce–O3,N3	2.689(11)	Ce–O3,N3	2.739(11)

a Refined cell parameters and agreement factors are *a* = 5.75284(19), *b* = 5.75620(15), and *c* = 12.71338(18) Å. *V* = 420.997(19) Å^3^. *R*_Bragg_ = 5.22% and *R*_wp_ = 1.34%.

b Estimated standard deviations in parentheses are shown once for each independent variable. The thermal parameters were refined anisotropically for the three anions; ellipsoids are shown on Figure 6. Average bond distances: Ta–O,N 2.090 Å and Ce–O,N 2.769 Å.

### Magnetic Properties

**Figure 10:**
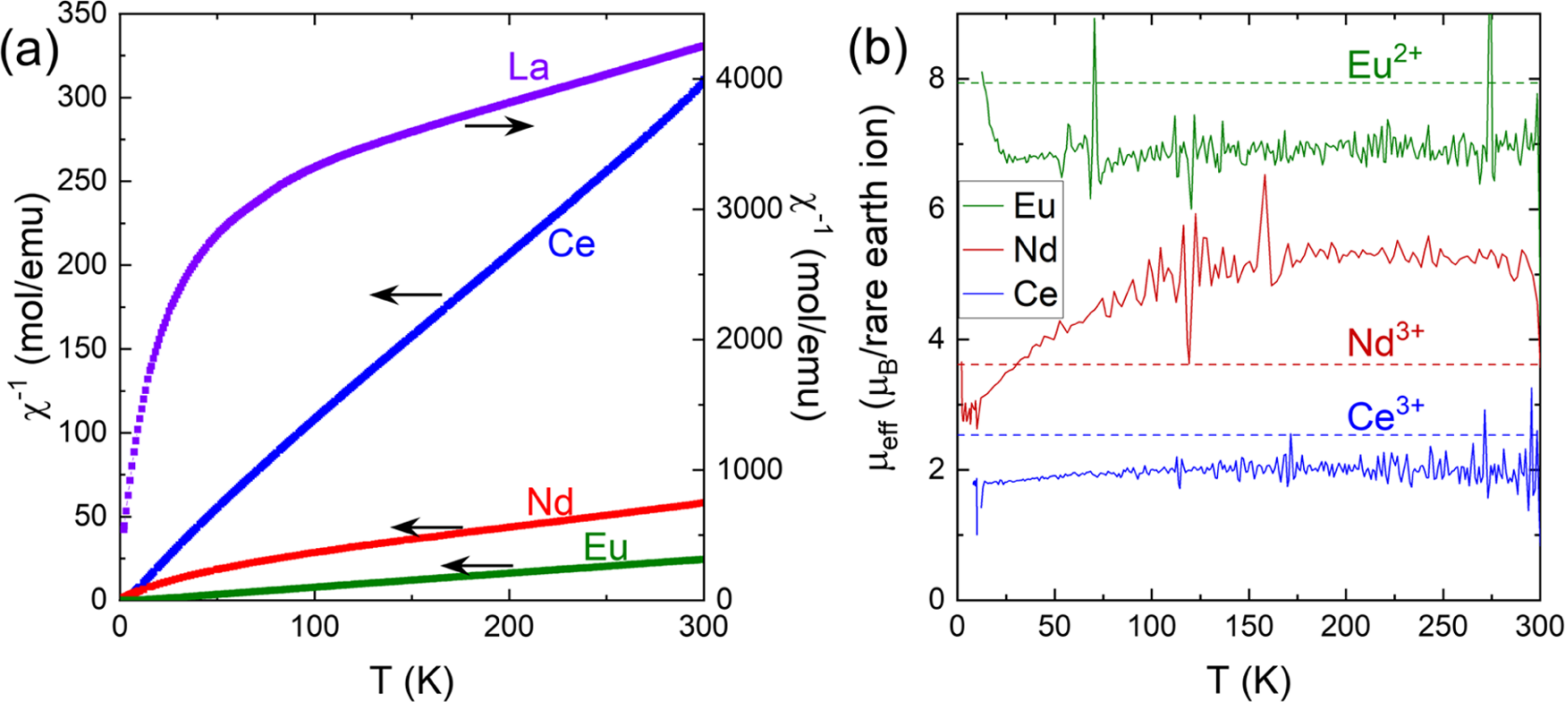
(a) Inverse susceptibility of La_2_TaO_1.31_N_2.69_, Ce_2_TaO_1.19_N_2.81_, Nd_2_TaO_1.46_N_2.54_, and Eu_2_TaO_2.80_N_1.20_, measured at 10 kOe (note the different scale, right axis, used for the La compound). (b) Temperature dependence of the effective paramagnetic moment obtained by the derivative of the inverse susceptibility as explained in the text.

The Ce, Nd, and Eu compounds display a Curie-like paramagnetic susceptibility at high temperature (*T* > 50 K) (Figure 10a), although deviations are clearly perceptible at least in Nd_2_TaO_1.46_N_2.54_, as typically found in rare earth compounds.^40^ La_2_TaO_1.31_N_2.69_ shows an extremely small paramagnetic susceptibility, in accordance with the nonmagnetic nature of La^3+^ and plausibly associated with the presence of Ta^4+^ or to traces of magnetic impurity. A convenient way to identify deviations from the common Curie behavior and to get insights into their physical origin is to plot the effective paramagnetic moment (μ_eff_), extracted from the measured susceptibility: . In Figure 10b, we show the μ_eff_ vs *T* plot, where μ_eff_ has been obtained through the thermal derivative of the inverse susceptibility: . It turns out that for Eu and Ce compounds, μ_eff_ is temperature-independent down to about 25K. The observed effective moments approach, although slightly smaller, to those expected for Eu^2+^ ions (^8^S_7/2_; *g*_J_ = 2) and Ce^3+^ (^2^F_5/2_, *g*_J_ = 6/7) (green and blue dashed lines in Figure 10b), which may indicate some overoxidation of Eu^2+^ and Ce^3+^. In fact, from the susceptibility of Ce_2_TaO_1.19_N_2.81_, we infer an effective paramagnetic moment of μ_eff_≃ 2.02 μ_B_/Ce, which could signal the partial appearance of Ce^4+^(J = 0), estimated to be around 37%, together with an accompanying fraction of Ta^4+^ (*J* = 1/2) for charge compensation. The presence of Ce^4+^ may be induced by the existence of nitrogen-rich regions created by the anion disorder, that will be balanced by oxide-rich regions where Ta^5+^ is reduced to Ta^4+^ according to the internal redox equilibrium Ce^3+^ + Ta^5+^⇔ Ce^4+^ + Ta^4+^.^4^ The observed effective paramagnetic moment of Eu_2_TaO_2.80_N_1.20_ is μ_eff_≃ 7.63 μ_B_/Eu, which assuming a coexistence of Eu^2+^ and Eu^3+^ would correspond to a concentration of Eu^3+^ of about 10%, in excellent agreement with the chemical analysis. The small magnetic moment in the broad and rather delocalized 5d^1^ orbitals of Ta^4+^ ions should lead to a minor contribution to the measured small susceptibility. In contrast, the Nd_2_TaO_1.46_N_2.54_ compound displays a conspicuous decrease of μ_eff_ on cooling below *T* ≃ 100 K. This is the common behavior of rare earths with an odd number of electrons in magnetically diluted systems, and it may result from crystal field effects or the presence of magnetic interactions, as discussed latter. For instance, the crystal field may break the degeneracy of the ground-state ^2S+1^L_J_ of the rare earth into various site symmetry-dependent Kramers doublets, with electron occupancy, and thus, the magnetic susceptibility will evolve with temperature. The presence of magnetic interactions may also result in a reduction of magnetic susceptibility. It follows that the origin of the observed temperature dependence of μ_eff_(*T*) cannot be, in general, univocally disentangled.^41^ On the other hand, the observation that in the high-temperature limit, the measured μ_eff_ is larger than the free ion Nd^3+^ value (^4^I_9/2_; *J* = 9/2, *g*_J_ = 8/11) remains intriguing. It could be tentatively attributed to some spin polarization of neighboring Ta^4+^ ions, as in Nd_2_TaO_1.46_N_2.54_, the proportion of this cation is the largest among the R_2_TaO_4–*x*_N_*x*_ series presented here.

**Figure 11:**
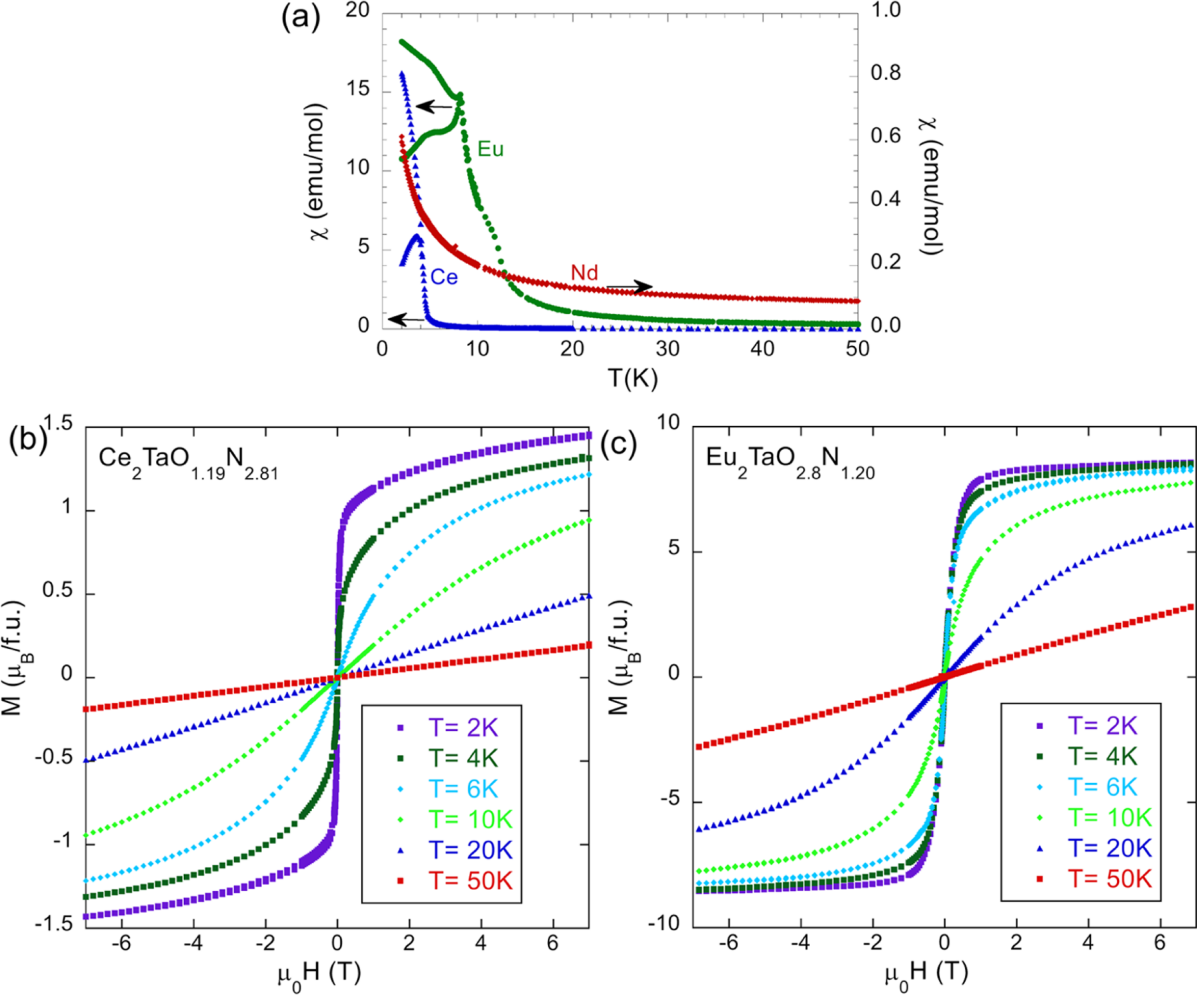
(a) Zero field-cooled/field-cooled magnetization vs temperature (measured under 25 Oe applied magnetic field) of Nd_2_TaO_1.46_N_2.54_ (right axis) and Ce_2_TaO_1.19_N_2.81_ and Eu_2_TaO_2.80_N_1.20_ (left axis). (b, c) Magnetization vs magnetic field measured at different temperatures for Ce_2_TaO_1.19_N_2.81_ and Eu_2_TaO_2.80_N_1.20_, respectively.

To get a deeper insight into the low-temperature spontaneous magnetic behavior of these compounds, we explored the low-field magnetic susceptibility (χ). In Figure 11a, we show χ(*T*) measured on heating under 25 Oe magnetic field after a zero-field cooling (ZFC) and field cooling (FC). A well-pronounced peak followed by a low-temperature hysteresis shows up in the ZFC-FC at ∼4K for Ce_2_TaO_1.19_N_2.81_ and ∼8K for Eu_2_TaO_2.80_N_1.20_, indicating the appearance of magnetic order in these compounds. In contrast, Nd_2_TaO_1.46_N_2.54_ does not display any hysteresis and thus no traces of magnetic order down to the lowest explored temperature (2K). To understand the origin of this hysteresis, we have measured the field-dependent magnetization. The obtained *M*(*H*) curves are depicted in Figure 11b,c. Data show a rapid upturn of magnetic moment under a low magnetic field at low temperatures characteristic of magnetic order with a ferromagnetic component. We have discarded that this upturn corresponds to a paramagnet at low enough temperature by plotting the magnetization vs *H*/*T* and checked that the *M*(*H*/*T*) curves at the lowest temperature (*T* < 15 K) do not scale (see Figure S3). Data collected at the lowest temperature reflect a lack of saturation, suggesting the coexistence of the remaining fraction of disordered spins in the samples.

The saturation magnetization (*M*_S_) inferred from data collected at the lowest temperature (2K) and largest field (7 T) indicates M_S_(Ce_2_TaO_1.19_N_2.81_) ∼ 0.7 μ_B_/Ce and M_S_(Eu_2_TaO_2.80_N_1.20_) ∼ 4.3 μ_B_/Eu. Both values are significantly smaller than those expected for fully collinear ferromagnetic orders: 2.14 μ_B_/Ce for Ce^3+^ and 1.35 μ_B_/Ce for the aforementioned 37% of Ce^4+^; 7 μ_B_/Eu for full Eu^2+^, and 6.3 μ_B_/Eu for 10% of Eu^3+^ (according to chemical analysis). This discrepancy between the expected (or the maximal) and observed values of the saturation magnetization indicates that a more complex ordering pattern could be at work or that the disorder in the system drives to magnetic frustration and to an only partially ordered magnetic structure.

**Figure 12:**
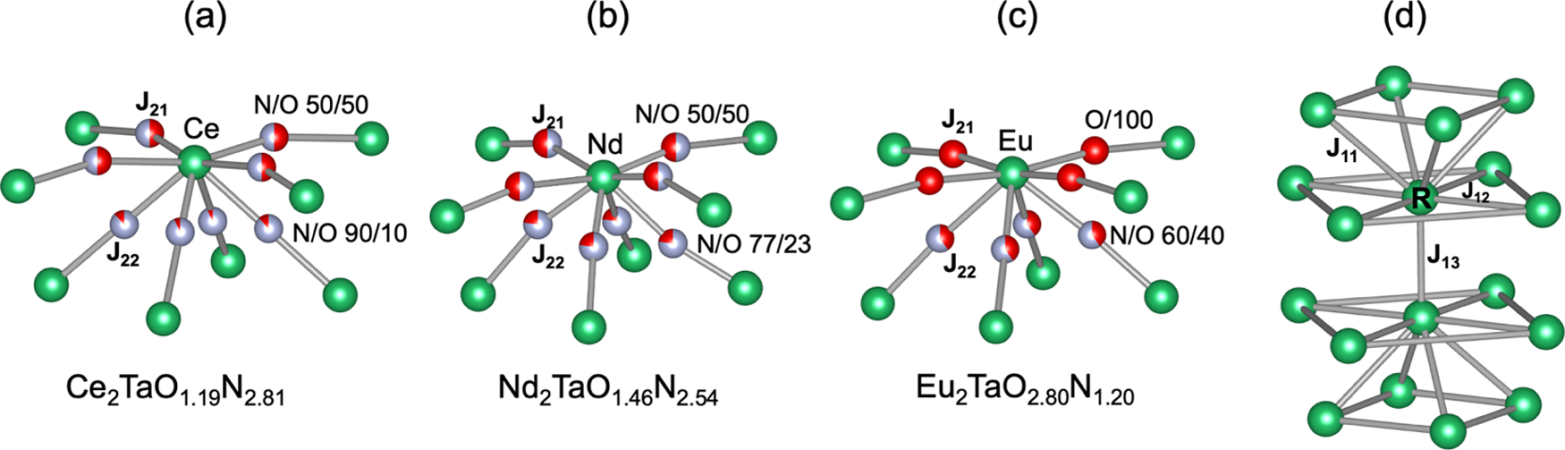
(a–c) Superexchange (J_21_, J_22_) and (d) direct exchange (J_11_, J_12_, J_13_) magnetic interactions in R_2_TaO_3-*x*_N_*x*_ compounds (R = Ce, Nd, Eu). Anion occupancies correspond to those determined from neutron diffraction for Ce_2_TaO_1.19_N_2.81_. For Nd_2_TaO_1.46_N_2.54_ and Eu_2_TaO_2.80_N_1.20_, the anion populations are those expected using PSCR (see the precedent section).

**Figure 13:**
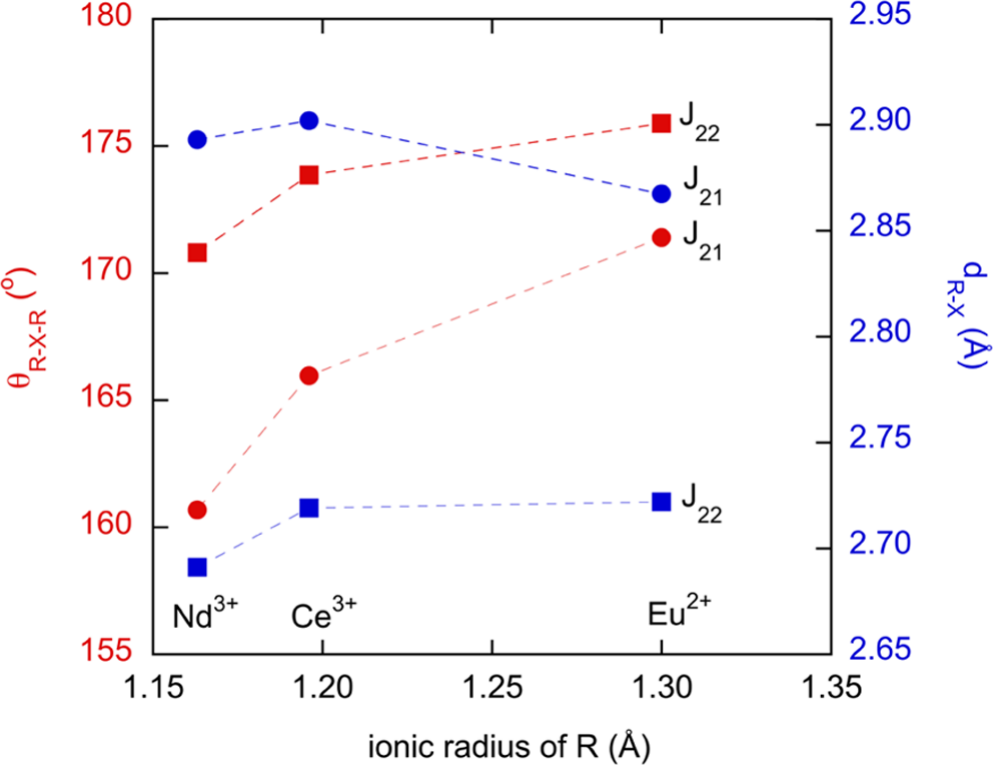
Average R–X–R angles and R–X (X = O,N) bond distances for superexchange interactions in Ce_2_TaO_1.19_N_2.81_ (from neutron diffraction data), Nd_2_TaO_1.46_N_2.54_, and Eu_2_TaO_2.80_N_1.20_ plotted against the ionic radii of Ce^3+^, Nd^3+^, and Eu^2+^, respectively, for CN = IX.^34^

At first sight, the lack of magnetic ordering in Nd_2_TaO_1.46_N_2.54_ may seem surprising as structural differences between the Nd, Ce, and Eu compounds are rather small. For the rare earth cations in the K_2_NiF_4_-type structure, there are eight superexchange pathways corresponding to the next nearest neighbors (NNN), four from the same NaCl-type layer (J_21_) and four from a neighbor layer (J_22_) (see Figure 12a–c).^42^ In these pathways, the changes of bond distances across the series are extremely moderate (≈1%), and the R–X–R bond angles change monotonically by ≈3% (171.41° for the Eu compound, 167.7° for Ce_2_TaO_1.19_N_2.81_ and 160.7° for Nd_2_TaO_1.46_N_2.54_) (Figure 13). On the other hand, any superexchange R–X–R magnetic interaction is expected to be stronger when increasing the covalency of bonds by reducing the electronegativity of the anions,^43^ and thus, a larger N/O ratio will reinforce the superexchange interactions. As illustrated in Figure 12, the N/O occupancy in the R–X–R pathways for Nd_2_TaO_1.46_N_2.54_ is similar to that of the Ce compound and larger than in the Eu compound, and still no magnetic order is observed in the Nd compound. From these two sets of data, we conclude that superexchange interactions do not appear to play a major role in the magnetic ordering of the rare earth ions in these compounds, which thus appear to be governed by direct R–R exchange interactions depicted in Figure 12d. In exchange-coupled Nd^3+^-Nd^3+^ units, crystal field and exchange interactions conspicuously combine to produce a singlet ground state, which is in sharp contrast with Ce^3+^-Ce^3+^ and Eu^2+^-Eu^2+^units where the ground state can be a triplet (see, for instance, Figures 31, 26, and 22, pages 64–69 in ref [^40^]). It follows that the effective magnetic moment of Nd^3+^ decreases when decreasing temperature, and no magnetic order develops in Nd_2_TaO_1.46_N_2.54_, which is completely different than the behavior of Ce_2_TaO_1.19_N_2.81_ and Eu_2_TaO_2.80_N_1.20_ compounds in agreement with our experimental observations.

## Conclusions

The new *n* = 1 Ruddlesden–Popper rare earth tantalum oxynitrides R_2_TaO_4-*x*_N_*x*_ (R = La, Ce, Nd, and Eu) are prepared using a solid-state reaction under N_2_ at temperatures between 1200 and 1700 °C starting with mixtures of R_2_O_3_, RN, Ta_3_N_5,_ and TaON. This is a versatile synthetic approach that allows to control the initial N/O ratio, a determining factor to stabilize the oxynitrides, by using different proportions of the four reactants while keeping the R/Ta = 2 stoichiometric ratio. The chemical analyses of the obtained oxynitrides La_2_TaO_1.31_N_2.69_, Ce_2_TaO_1.19_N_2.81_, Nd_2_TaO_1.46_N_2.54_, and Eu_2_TaO_2.80_N_1.20_ indicate a decrease of the N/O ratio during the synthesis that leads to an excess of oxygen with respect to the ideal stoichiometry R_2_TaON_3_, corresponding to the oxidation states of the cations R^3+^ and Ta^5+^. The lower nitrogen content is formally charge-compensated by the reduction of europium to the divalent state or of Ta^5+^ to Ta^4+^.

The La, Ce, and Nd compounds show a tilted superstructure with cell parameters √2*a*_0_ × √2*a*_0_ × *c*_0_ (where *a*_0_ and *c*_0_ are the parameters of the *I*4/*mmm* K_2_NiF_4_ aristotype) with the *Pccn* space group. In contrast, the europium compound shows additional doubling of the *c* axis, with parameters √2a_0_ × √2a_0_ × 2c_0_, and crystallizes in the *I*4_1_/*acd* space group. The observed different crystal chemistry in the europium compound is a consequence of the near total reduction of this rare earth to Eu^2+^. The neutron diffraction study of Ce_2_TaO_1.19_N_2.81_ shows that the equatorial sites of the tantalum octahedra have an occupancy of nearly 100% nitrogen, whereas the axial sites are occupied by 50% of each anion. This anion distribution is in excellent agreement with the prediction of Pauling’s second crystal rule that leads to the bond strength sums of 2.94 and 2.47 for the equatorial and axial sites, respectively. According to this prediction, a similar anion order is expected for the trivalent rare earth *n* = 1 Ruddlesden–Popper oxynitrides of La and Nd. For the Eu^2+^ compound, the corresponding calculated sums are 2.55 and 1.94; hence, a population of 50/50 O/N in the equatorial sites and 100% O in the axial sites is predicted. The Ce and Eu compounds display some magnetic order at low temperatures with a ferromagnetic component. In contrast, the Nd oxynitride does not show any fingerprint of magnetic order but remains paramagnetic down to the lowest temperature explored (2 K), consistent with the temperature-independent effective magnetic moment observed in the former and a low-temperature suppression in the latter, which we attribute to the combined effect of a temperature-dependent change of electron occupancy in the crystal-field split Kramers doublets and exchange interactions producing a singlet ground state. The new *n* = 1 Ruddlesden–Popper compounds reported in this paper expand the structural diversity of the family of perovskite oxynitrides opening avenues to search new materials in this group of solids. Post-treatments of the R_2_TaO_4–*x*_N_*x*_ samples in strongly nitriding atmospheres such as NH_3_ would plausibly increase the nitrogen contents with concomitant oxidation of the cations Eu^2+^ and Ta^4+^, and new applications as dielectric materials or as visible light photocatalysts in different reactions may emerge.

## Supporting Information Available

Electron diffraction patterns of La_2_TaO_1.31_N_2.69_ and Nd_2_TaO_1.46_N_2.54_, refinement of synchrotron X-ray powder diffraction data of Ce_2_TaO_1.19_N_2.81_, and magnetization vs μ_0_*H*/*T* curves of Ce_2_TaO_1.19_N_2.81_ and Eu_2_TaO_2.80_N_1.20_ (PDF)
