# Supplementary material for: Anionic and Magnetic Ordering in Rare Earth Tantalum Oxynitrides with an n = 1 Ruddlesden–Popper Structure
Source: Chem Mater. 2024 May 8;36(10):5160–71. doi: 10.1021/acs.chemmater.4c00533 (PMC11138149; doi:10.1021/acs.chemmater.4c00533)
Supplement: Supplementary file 1 — cm4c00533_si_001.pdf [file cm4c00533_si_001.pdf]

Supplementary Information

Anionic and magnetic order in rare earth tantalum oxynitrides with n=1 Ruddlesden Popper structure

Jhonatan R. Guarín, Carlos Frontera, Judith Oró-Solé, Bastian Colombel, Clemens Ritter, François Fauth, Josep Fontcuberta and Amparo Fuertes**

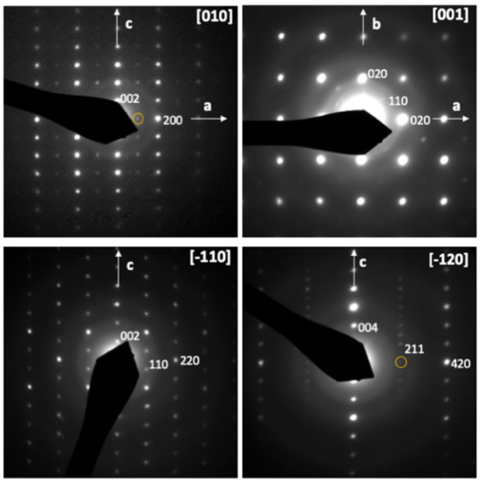

Figure S1. Electron diffraction patterns of $La_2TaO_{1.31}N_{2.69}$. Yellow circles indicate multiple diffraction reflections.

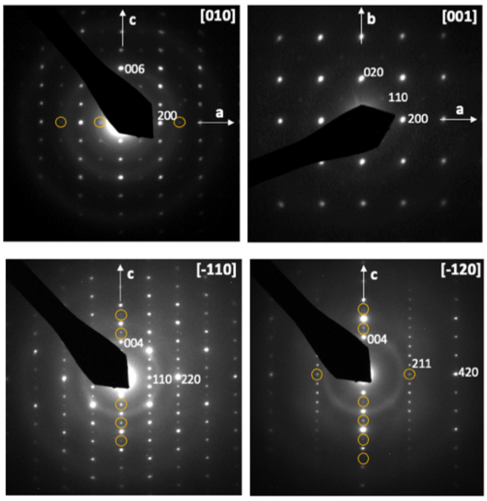

Figure S2. Electron diffraction patterns of $Nd_2TaO_{1.46}N_{2.54.}$ Yellow circles indicate multiple diffraction reflections.

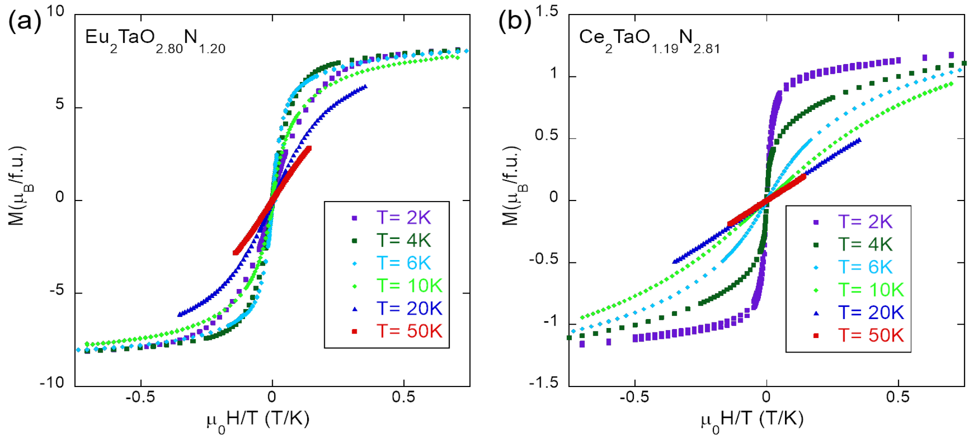

Figure S3. Magnetization plotted in front of μ_0H/T at different temperatures for a) $Eu_2TaO_{2.80}N_{1.20}$ and b) $Ce_2TaO_{1.19}N_{2.81}$. The lack of scaling of the curves proves that magnetization does not follow a Langevin nor a Brillouin function that describe paramagnetism.

Table S1. Summary of the *Pccn* model refined against room temperature synchrotron X-ray powder diffraction data for $Ce_2TaO_{1.19}N_{2.81}$ (λ=0.4137 Å). Refined cell parameters and agreement factors are: a=5.70500(5), b=5.71182(4), c=12.61280(7) Å. V= 411.000(5) $Å^3$. R_{Bragg}= 5.54%, R_{wp}= 9.68 %. [a]

Atom	*Site*	x	y	z	B($Å^2$)	Occupancy
Ce	*8e*	0.4958(4)	0.0089(4)	0.1428(4)	0. 565(9)	1
Ta	*4a*	0	0	0	0.996(16)	1
O1/N1	*8e*	0.03090	0.04010	0.17037	2.29(15)	0.498/0.502
O2/N2	4c	0.25	0.25	0.4800	2.29	0.070/0.930
O3/N3	*4d*	0.25	0.75	0.0011	2.29	0.124/0.876

Bond	d(Å)	Bond	d(Å)	Bond	d(Å)
Ta-O1,N1	2.168x2	Ta-O2,N2	2.034x2	Ta-O3,N3	2.018x2
Ce-O1,N1	2.381(1)	Ce-O1,N1	2.604(2)	Ce-O1,N1	2.681(2)
Ce-O1,N1	3.078(3)	Ce-O1,N1	3.159(3)	Ce-O2,N2	2.587(2)
Ce-O2,N2	2.843(2)	Ce-O3,N3	2.701(2)	Ce-O3,N3	2.711(2)

[a] Estimated standard deviations in parentheses are shown once for each independent variable. The temperature factors were common for the three anions sites. Average bond distances: Ta-O,N 2.073 Å; Ce-O,N 2.749 Å.
